# Phasevarions in *Haemophilus influenzae* biogroup *aegyptius* control expression of multiple proteins

**DOI:** 10.1128/spectrum.02601-23

**Published:** 2023-12-06

**Authors:** Greg Tram, Freda E.-C. Jen, Zachary N. Phillips, John F. Lancashire, Jamie Timms, Jessica Poole, Michael P. Jennings, John M. Atack

**Affiliations:** 1 Institute for Glycomics, Griffith University, Gold Coast, Queensland, Australia; 2 School of Environment and Science, Griffith University, Gold Coast, Queensland, Australia; Center of Innovative and Applied Bioprocessing, Mohali, Punjab, India

**Keywords:** phase variation, phasevarion, *Haemophilus influenzae *biogroup *aegyptius*

## Abstract

*Haemophilus influenzae* biogroup *aegyptius* is the causative agent of Brazilian purpuric fever (BPF), an invasive disease with high mortality, that sporadically manifests in children previously suffering conjunctivitis. Though strains of *H. influenzae* biogroup *aegyptius* that cause BPF are distinct from conjunctival strains, individual factors that directly correlate with the manifestation of BPF have not been conclusively identified. Phase variation is the rapid and reversible switching of gene expression, and is typically associated with bacterial surface proteins. However, over the last ~15 years, the importance of phase-variable, cytoplasmic DNA methyltransferases in bacterial gene regulation has been reported. Variable expression of a DNA methyltransferase results in genome-wide methylation differences and regulation of multiple genes by epigenetic mechanisms. These systems are called phasevarions (phase-variable regulons). An analysis of the *modA* alleles present in genomes of *H. influenzae* biogroup *aegyptius* demonstrated that all BPF causing isolates encoded the uncharacterized *modA16* allele. The majority of non-BPF strains encoded the ModA13 allele, previously unstudied in *Haemophilus* spp. We demonstrate biphasic ON-OFF switching of both ModA13 and ModA16 and determine the distinct DNA motifs methylated by these enzymes. Differential methylation in isogenic ModA ON-OFF strain pairs results in the control of distinct phasevarions. These phasevarions include several previously characterized virulence factors. Comparison of these isogenic ON-OFF pairs of strains encoding ModA13 or ModA16 determined that switching of these phasevarions did not alter serum resistance, a key characteristic of BPF causing strains of *H. influenzae* biogroup *aegyptius*. This study provides new information about how key virulence determinants are regulated in *H. influenzae* biogroup *aegyptius*.

**IMPORTANCE:**

*Haemophilus influenzae* biogroup *aegyptius* is a human-adapted pathogen and the causative agent of Brazilian purpuric fever (BPF), an invasive disease with high mortality, that sporadically manifests in children previously suffering conjunctivitis. Phase variation is a rapid and reversible switching of gene expression found in many bacterial species, and typically associated with outer-membrane proteins. Phase variation of cytoplasmic DNA methyltransferases has been shown to play important roles in bacterial gene regulation and can act as epigenetic switches, regulating the expression of multiple genes as part of systems called phasevarions (phase-variable regulons). This study characterized two alleles of the ModA phasevarion present in *H. influenzae* biogroup *aegyptius*, ModA13, found in non-BPF causing strains and ModA16, unique to BPF causing isolates. Phase variation of ModA13 and ModA16 led to genome-wide changes to DNA methylation resulting in altered protein expression. These changes did not affect serum resistance in *H. influenzae* biogroup *aegyptius* strains.

## INTRODUCTION

*Haemophilus influenzae* biogroup *aegyptius* emerged as the causative agent of an invasive disease known as Brazilian purpuric fever (BPF). Similar in symptomatic presentation to meningococcal meningitis, cases of BPF were usually preceded by purulent conjunctivitis, and were characterized by a rapid and severe onset of disease, with high mortality (1). Most cases of BPF occurred in the 1980s and early 1990s in Brazil (1). Outside Brazil, one case was described in Israel in 1986, two cases in Australia in 1986, and a single case in the United States in 1993 (1). Considered a subspecies of *H. influenzae,* the population of invasive BPF causing *H. influenzae* biogroup *aegyptius* strains is clonal and is genetically distinct from those which only cause conjunctivitis (2). It remains unknown what gave rise to the unusual invasive disease seen in some strains of *H. influenzae* biogroup *aegyptius*, or what differentiates BPF causing strains from non-BPF strains. Early analysis of BPF vs non-BPF causing strains demonstrated an increased serum resistance in BPF causing strains, but the mechanism of BPF strain resistance to lysis by human serum was not defined (3). *H. influenzae* biogroup *aegyptius* strains encode a number of lineage-specific adhesins (4), with similarity to known non-typeable *Haemophilus influenzae* (NTHi) virulence factors such as the HMW1/2 proteins (5), and multiple novel autotransporter protein adhesins (4). Three of these adhesins, Las, TabA1, and HadA1, all showed variable expression when interacting with human cells, or during an infant rat model of *H. influenzae* biogroup *aegyptius* disease (6). A homolog of the Las protein, named Lav, has been demonstrated to be required for adherence to host cells in NTHi (7). *H. influenzae* biogroup *aegyptius* strains also encode several lineage-specific fimbriae, well-characterized bacterial virulence determinants (4). However, these previous analyses demonstrated that no single factor is involved in BPF manifestation. Many of these lineage-specific adhesins present in *H. influenzae* biogroup *aegyptius* contain simple DNA sequence repeat (SSR) tracts (4), indicative that phase variation may play a role in disease progression and BPF manifestation.

Phase variation is the rapid and reversible switching of gene expression (8, 9), often mediated by SSRs. Many virulence factors in *H. influenzae* are encoded by phase-variable genes (10, 11). SSR tracts are short, repetitive sequences of DNA, and are a hallmark of phase variation (12 – 14); gain or loss of individual repeat units in SSR tracts during DNA replication results in ON-OFF switching of gene expression. Phase variation serves as a contingency strategy that generates a diverse bacterial population containing multiple phenotypic variants (12, 15). Recent studies have demonstrated that almost 20% of all cytoplasmically located Type III DNA methyltransferases (Mod proteins) are potentially phase-variable, as their encoding genes (*mod*) contain SSR tracts (16). Differential expression of cytoplasmic methyltransferases due to ON-OFF switching leads to variable genome-wide methylation within a population of bacteria and differential expression of multiple genes by epigenetic mechanisms. These systems are known as phasevarions (phase-variable regulons) (15, 17, 18). In every described case, phasevarions control genes involved in bacterial pathobiology, and many control expression of current and putative vaccine candidates, and regulation of processes resulting in differential susceptibility to antibiotics (15, 17, 19).

ModA DNA methyltransferases, present in multiple *Haemophilus* and *Neisseria* species (20 – 22), control multiple different phasevarions. There are currently 22 different *modA* allelic variants identified (20, 21, 23). All characterized *modA* alleles that phase-vary do so through changes in length of a locus-associated AGCC_(n)_ SSR tract, located in the open reading frame of the *modA* gene (18, 20 – 22). *modA* genes are highly conserved (over 90% sequence identity) in their 5′ and 3′ regions, with *modA* allelic variants delineated based on their highly variable central region (23), which encodes the target recognition domain (TRD). The TRD dictates the DNA sequence recognized and methylated by the ModA protein. Therefore, different TRDs result in a different DNA sequence being recognized and methylated by the encoded ModA protein, and therefore, a different suite of genes is regulated. Through this mechanism, different ModA alleles regulate different phasevarions. Phase-variable expression of multiple ModA allelic variants has been shown to have global effects on bacterial phenotype, including regulating expression of outer-surface proteins (20, 22) and controlling key phenotypes related to virulence and pathogenicity of *H. influenzae* (18, 20, 24 – 26) and *Neisseria* spp. (22, 27).

Our examination of multiple *H. influenzae* biogroup *aegyptius* strains with closed annotated genomes (28) demonstrated the presence of two ModA alleles previously undescribed in *Haemophilus* spp.: the ModA13 allele, which has been shown to regulate a phasevarion in *Neisseria gonorrhoeae* (22), and the ModA16 allele, which has not been characterized in *H. influenzae* biogroup *aegyptius*, but had been observed before in NTHi isolates. We therefore hypothesized that these two allelic variants of ModA may regulate virulence phenotypes in BPF causing isolates of *H. influenzae* biogroup *aegyptius*.

## RESULTS

### ModA13 and ModA16 are phase-variable DNA methyltransferases

To determine how prevalent the *modA13* and *modA16* variants are in *H. influenzae* biogroup *aegyptius,* we conducted an examination of all 10 *H. influenzae* biogroup *aegyptius* strains with a closed annotated genome available in NCBI GenBank (see Table 1). This determined that the *modA16* allele was encoded in four of these strains, (strains F1946, F3028, F3037, and F3031) (2, 28), all isolated in Brazil in the 1980s from patients presenting with BPF (2). The *modA16* gene in all four of these strains contained variable numbers of AGCC_(n)_ repeats, strongly indicative of phase-variable expression (28). The remaining six strains, either isolated from non-BPF patients or with no information on site of isolation, did not encode the *modA16* allele. A single strain, F3043, encoded the *modA7* allele, previously identified in NTHi (23). Interestingly, the *modA7* allele in strain F3043 contained an AGCC_(n)_ repeat tract, which is the first identified instance of the *modA7* allele being potentially phase-variable; all previously identified *modA7* alleles did not contain an AGCC_(n)_ SSR tract, and were therefore unable to phase-vary (20, 21, 23). Strains F3047 (4), F3052 (28), NCTC8134, NCTC8502, and FDAARGOS_1478 (all unpublished) encoded the *modA13* allele, which has previously been characterized in *Neisseria gonorrhoeae* (22).

**TABLE 1:** Analysis of *modA* alleles in all strains in GenBank identified as *H. influenzae* biogroup *aegyptius* with a closed annotated genome^*a*^

Strain	Source	Disease sequelae	Genome accession number	*modA* allele	Reference
F1946	Skin (petechiae)	BPF	CP043770.1	*modA16*	(28)
F3028	CSF^*b*^	BPF	CP043771.1	*modA16*	(28)
F3037	Blood	BPF	CP043772.1	*modA16*	(28)
F3031	Brazilian purpuric fever	BPF	FQ670178	*modA16*	(4)
F3043	Conjunctiva	Non-BPF	CP043811.1	*modA7*	(28)
F3052	Conjunctiva	Non-BPF	CP043810.1	*modA13*	(28)
F3047	Conjunctiva	Non-BPF	FQ670204	*modA13*	(4)
NCTC8134	Not available	Not available	LR134395.1	*modA13*	Unpublished
NCTC8502	Not available	Not available	LS483429.1	*modA13*	Unpublished
FDAARGOS_1478	Not available	Not available	CP082857.1	*modA13*	Unpublished

^*a*^ The ModA16 allele was only found in BPF isolates.

^*b*^ Cerebrospinal fluid.

Further analysis of closed annotated genomes in NCBI GenBank using strains identified as *Haemophilus influenzae* determined that the *modA16* allele was only present in the closed genomes of three additional strains: M17648 (accession number CP031241.1), M14951 (accession number CP031244.1), and M12125 (accession number CP031237.1). All three of these strains were isolated from patients presenting with meningitis. The only other *H. influenzae* strain identified as encoding a *modA16* allele was strain ATCC 9007, with this sequence being a direct submission of the TRD region of the *modA* allele (approximately 600 bp) from this strain (accession number EF068010.1) (23).

**Fig 1:**
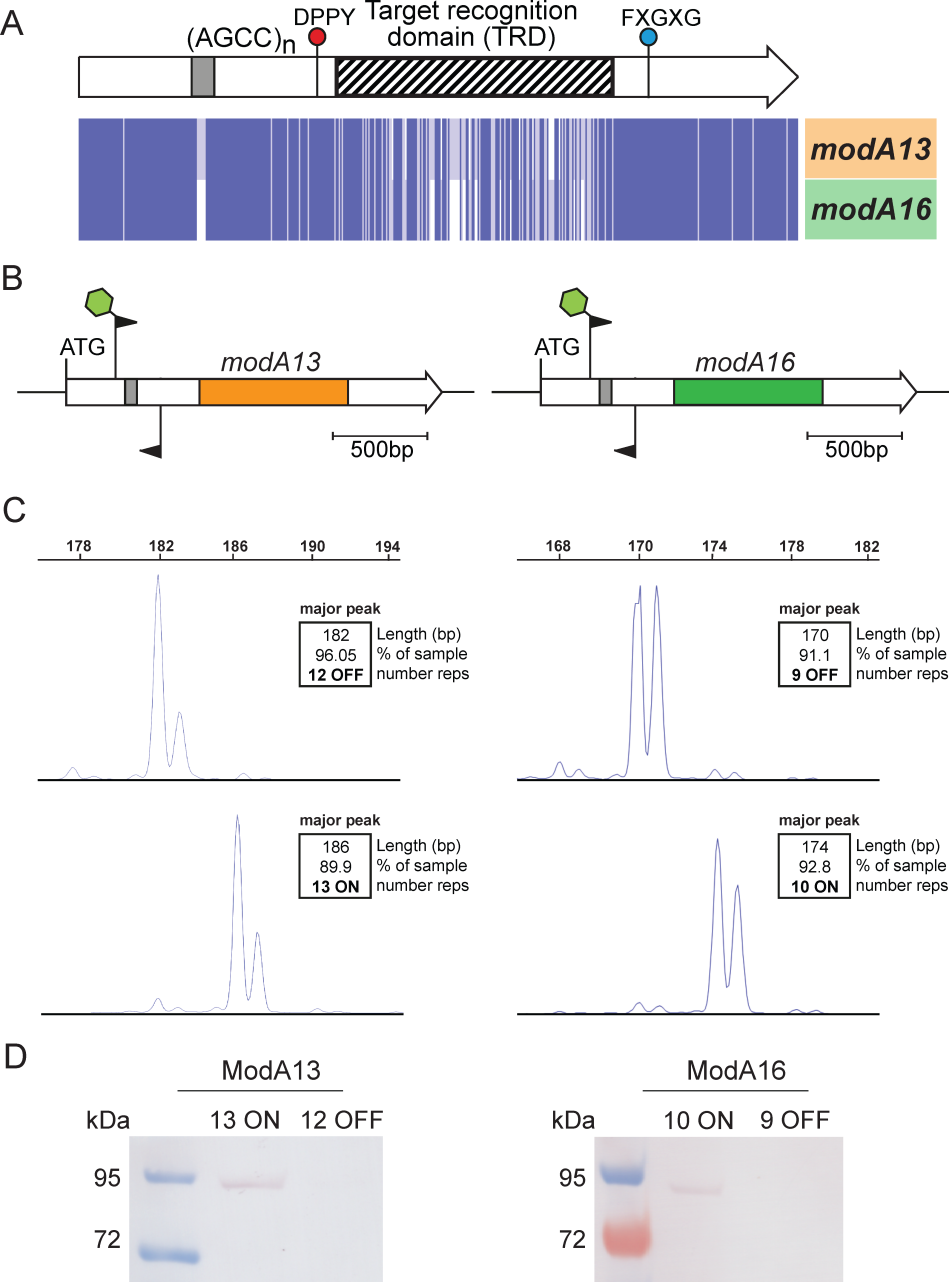
Demonstration of the biphasic switching in *modA13* and *modA16*. (**A**) Illustration of modA gene; regions in white at the 5´ and 3´ ends of the gene are highly conserved between alleles (>95% nucleotide identity), whereas the central TRD, represented by a hatched box, is highly variable (<25% nucleotide identity between alleles). The AGCC_(n)_ repeat tract is represented by a gray box toward the 5´ end of the gene. Conserved regions encoding the catalytic domain DPPY and substrate binding domain FXGXG are also highlighted. The nucleotide alignment specifically illustrating the *modA13* and *modA16* genes highlights the highly variable central domain. Alignment was carried out using full-length DNA sequences of *modA13* and *modA16* using Muscle and visualized in JalView overview feature. Each blue line represents one nucleotide; (**B**) schematic representation of the *modA13* and *modA16* genes, highlighting the sites for 6-carboxyfluorescein (FAM)-labeled primers used for fragment analysis. The white regions represent conserved regions (>95% nucleotide identity) between all *modA* genes, with the orange (*modA*13) or green (*modA*16) box representing the highly variable TRD; (**C**) fragment length analysis traces showing populations of F3052 where *modA13* ON AGCC_(13)_ at 91.82% enrichment for an SSR tract length of 13 repeats and modA13 OFF AGCC_(12)_ at 96.83% enrichment for an SSR tract length of 12 repeats. Strain F3037 populations of *modA16* ON AGCC_(10)_ repeats show 92.83% enrichment for an SSR tract length of 10 repeats and that *modA16* OFF AGCC_(9)_ repeats show 91.10% enrichment for an SSR tract length of nine repeats; (**D**) Western blot using anti-ModA antisera against whole cell lysates of ModA13 and ModA16 ON-OFF strain pairs demonstrating that the ModA protein is only expressed in strains enriched for a number of 10 AGCC repeats that lead to the gene being in-frame (ON). Full Coomassie-stained gels and anti-ModA Western blots are shown in Fig. S1.

The strains chosen to characterize the ModA13 and ModA16 phasevarions in *H. influenzae* biogroup *aegyptius* were strain F3052, encoding the *modA13* allele, and strain F3037, encoding the *modA16* allele. These strains were chosen as both wild-type strains contained AGGC tract lengths in their respective *modA* genes that would strike a balance between ease of isolation of variants and stability when grown *in vitro*. Analysis of the nucleotide sequences of these two alleles demonstrated highly conserved 5′ and 3′ regions, and a highly variable central TRD region (Fig. 1A), as with our previous analyses of *modA* genes (16, 20 – 23). Our previous whole genome sequencing using these strains demonstrated that the *modA13* allele in strain F3052 contained 12 AGCC repeats, and the *modA16* allele in strain F3037 contained 10 AGCC repeats (Fig. 1B) (28). In order to study the effect of ModA13 and ModA16 methyltransferase phase variation on the phenotype of *H. influenzae* biogroup *aegyptius,* we carried out single colony enrichment to isolate ON and OFF variants which were approximately 90% pure for a single repeat tract length that would be either in-frame, and expressed (ON), or out of frame, and not expressed (OFF). This resulted in isolation of isogenic strain pairs in F3052 for *modA13* that were either 13 AGCC repeats ON (89.91% 13 repeats) or 12 AGCC repeats OFF (96.05% 12 repeats) (Fig. 1C), and in strain F3037 for *modA16* that were 10 AGCC repeats ON (92.83% 10 repeats) or nine repeats OFF (91.10% nine repeats) (Fig. 1C). A Western blot using anti-ModA antisera (20) demonstrated that a ModA protein band was only detected in the strains predicted to be ON—for ModA13 in strain F3052, the strain containing 13 AGCC repeats, and for ModA16 in strain F3037 the strain containing 10 AGCC repeats (Fig. 1D). ModA expression was not detected in either of the strains where the respective *modA* gene contained a number of repeats predicted to be OFF (Fig. 1D).

ModA13 (NgoAXII) was originally identified and studied in *Neisseria gonorrhoeae,* where it was demonstrated to regulate a phasevarion (22). This same paper also demonstrated a methylation specificity of AGAA^(m6)^ **A** using restriction-inhibition assays (22). A subsequent study of ModA13 determined methylation of a different target sequence by this enzyme, GCAG^(m6)^ **A**, carried out using the gold standard for determination of methyltransferase specificity, Pacific Biosciences (PacBio) Single-Molecule, Real-Time (SMRT) sequencing and methylome analysis (29). Due to this contradictory data, we sought to determine the correct ModA13 specificity, and the specificity of ModA16, using PacBio SMRT sequencing and methylome analysis using genomic DNA isolated from our ON and OFF enriched *modA13* and *modA16* variants (Fig. 1D).

The ModA13 ON-OFF pair of strain F3052 demonstrated an identical methylome (full methylomes of both strains in Supplementary Data 1) except for the motif 5′-GCAG^(m6)^ **A**, which was only detected in the ModA13 ON variant. This motif was detected as methylated at 99.9% of these sites in the ModA13 ON strain, but not detected at all in the ModA13 OFF strain. This motif matched that seen previously in *N. gonorrhoeae* using PacBio SMRT sequencing and methylome analysis (29) and not the AGAA^(m6)^ **A** motif initially described (22). In the sequence analyzed in this paper, there is a GCAGA motif overlapping the predicted AGAAA motif (22).

In strain F3037 encoding ModA16, the only motif present in the ModA16 ON variant which was not detected in the corresponding ModA16 OFF variant was 5′-GGRC^(m6)^ **A** (where R is either an A or G; Supplementary Data 1). This motif was detected as methylated at 94.5% of these sites in the ModA16 ON strain, but not detected at all in the ModA16 OFF strain. To confirm that this was the sequence ModA16 recognized and methylated, the *modA16* gene was cloned and heterologously over-expressed in *Escherichia coli* BL21 from the enriched ModA16 ON variant. We have previously demonstrated that heterologous over-expression of Type III Mod proteins in *E. coli* is suitable for determining methyltransferase specificity (16, 30, 31) and that the motifs detected by heterologous over-expression match the motif detected when the methyltransferase is expressed in the original strain (19, 30). Expression of ModA16 in BL21 was confirmed using an anti-ModA blot (Fig. S2). As seen in genomic DNA of F3037, SMRT sequencing and methylome analysis using plasmid DNA from a strain over-expressing ModA16 detected both 5′-G^(m6)^ **A**TC (Dam) and 5′-GGRC^(m6)^ **A**, but only detected Dam 5′-G^(m6)^ **A**TC in a BL21 strain containing pET28a only, confirming the motif specificity of ModA16 (Supplementary Data 1).

### ModA13 and ModA16 phase variation results in gross differences in outer-membrane protein (OMP) composition

**Fig 2:**
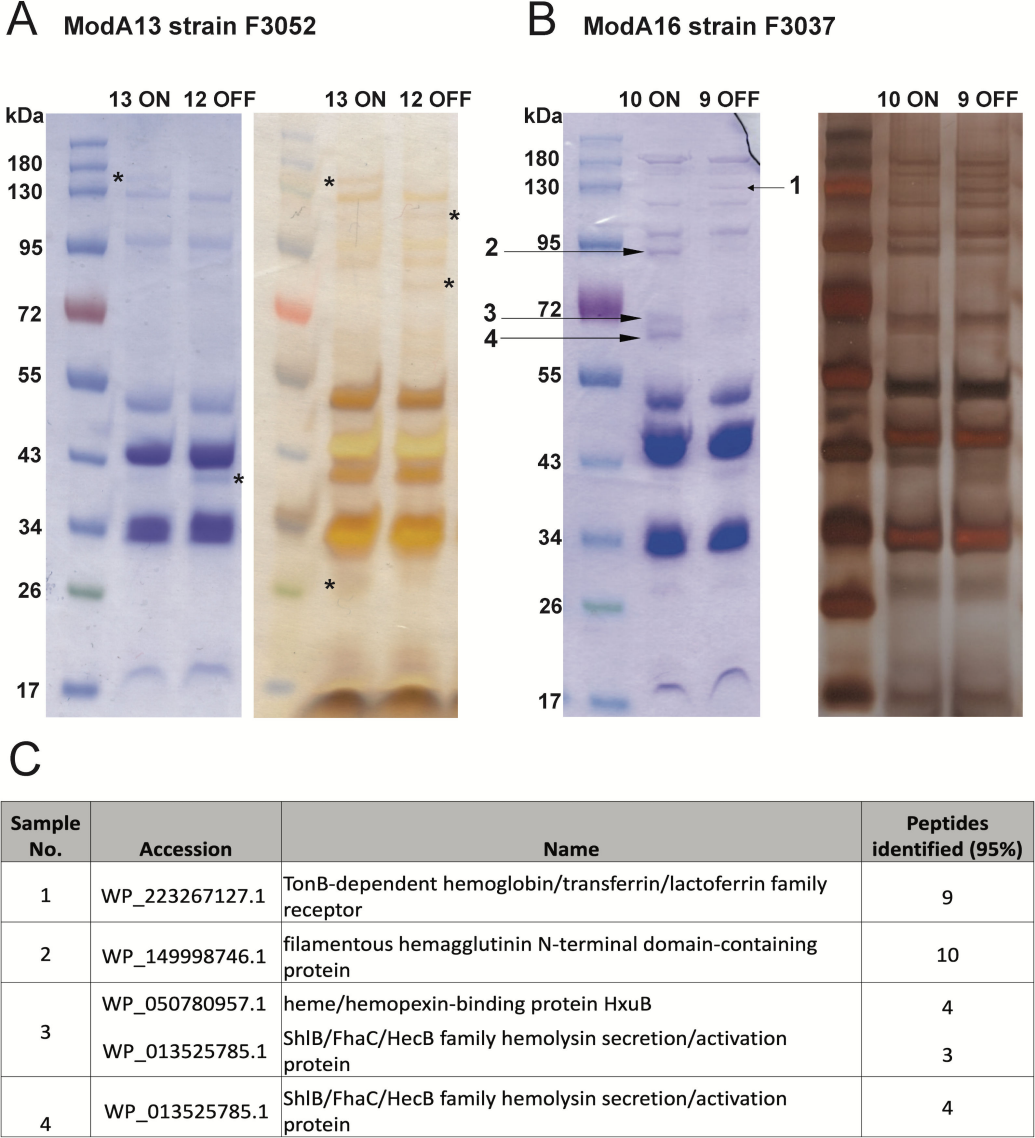
ModA13 and ModA16 phase variation results in differential expression of proteins in the outer membrane. Phase-variable expression of ModA13 and ModA16 alters gross difference in protein content of the outer membrane assessed with Coomassie blue staining and silver staining. (**A**) Bands with differences commensurate with ModA13 ON-OFF status are marked with a *. (**B**) Bands marked with an arrow (annotated as bands 1–4) that showed differences commensurate with ModA16 ON-OFF status were cut out of the Coomassie-stained gel and identified using mass spectrometry. (**C**) The proteins identified via mass spectrometry from the bands (samples 1 to 4) removed from the ModA16 SDS-PAGE gel.

Following confirmation that both ModA13 and ModA16 are active phase-variable DNA methyltransferases, we aimed to determine if ModA phase variation led to observable protein expression at the bacterial cell surface. We investigated the changes to outer-membrane protein expression as a result of ModA13 and ModA16 phase variation using our enriched ON-OFF pairs for each model strain. Following enrichment of outer-membrane proteins from bacteria using sarkosyl, several OMPs showed significantly altered expression levels when assessed using Coomassie-stained SDS-PAGE gels (Fig. 2A and B). Comparisons of the ModA13 ON and OFF OMP samples showed several proteins with differential expression in the Coomassie-stained gel (highlighted with * in Fig. 2A).

In the ModA16 ON vs OFF OMP samples, four protein bands showed prominent changes to expression levels in ModA16 ON and ModA16 OFF strains detectable by Coomassie blue staining. These bands were excised for liquid chromatography with tandem mass spectrometry (LC-MS/MS) protein identification. Sample 1 was a protein band at ~140 kDa and was found to be present at a lower level in ModA16 ON (i.e., upregulated in ModA16 OFF; Fig. 2B). The protein marked as sample 1 was identified as being a TonB-dependant receptor for hemoglobin, transferrin, and lactoferrin (Fig. 2B). The proteins marked as sample 2 (~100 kDa), sample 3 (~80 kDa), and sample 4 (~70 kDa) were bands which exhibited a significantly higher intensity in ModA16 ON (Fig. 2B). The most abundant protein in sample 2 was identified as containing a filamentous hemagglutinin N-terminal domain-containing protein. Sample 4 (Fig. 2A) was identified as containing a protein likely to be FhaC (Fig. 2C), the transporter for filamentous hemagglutinin.

### Phase variation of ModA13 and ModA16 results in whole cell differential protein expression

Following identification of specific OMPs with potential roles in pathogenesis, we sought to determine the impact of each distinct methyltransferase on genome-wide expression variations. To achieve this, we used sequential window acquisition of all theoretical mass spectra (SWATH-MS) to determine relative quantitative changes in protein levels in our enriched ModA13 and ModA16 ON-OFF pairs.

Proteomic analysis of the ModA13 ON-OFF identified a total of 263 proteins, covering 17% of the total proteome. Of these proteins identified, 208 exhibited statistically significant changes to expression due to ModA13 expression (adjusted *P*-value <0.05). Forty-nine of these proteins demonstrated a ≥1.5-fold change in abundance; 13 proteins were upregulated and 36 proteins were downregulated in response to ModA13 expression (Table 2). Proteins upregulated in response to ModA13 expression included proteins involved in cell division, protein assembly, and metabolism. The restriction enzyme (R) subunit from a Type I R-M system (1.55-fold) and an outer-membrane protein OmpH (1.67-fold) were also upregulated. Proteins downregulated due to ModA13 include primarily ribosomal proteins and those involved in metabolism (Table 2).

**TABLE 2:** Differential regulation of protein expression by the ModA13 phasevarion (>1.5-fold)

Accession	Protein	Fold change (ON vs OFF)	*p*-value
Down regulated in ModA13-ON			
WP_005632756.1	50S ribosomal protein L23	2.5	1.82 × 10^−7^
WP_005650452.1	Stringent starvation protein A	2.4	2.79 × 10^−6^
WP_006995958.1	Phenylalanine—tRNA ligase subunit beta	2.3	≤1.00 x 10^−17^
WP_150007840.1	Aminopeptidase N	2.1	3.33 × 10^−9^
WP_006995201.1	Aspartate ammonia-lyase	2	4.09 × 10^−5^
WP_006995540.1	50S ribosomal protein L21	2	6.06 × 10^−8^
WP_006996519.1	Thioredoxin-disulfide reductase	2	2.89 × 10^−6^
WP_150007547.1	Methionine—tRNA ligase	1.8	3.27 × 10^−4^
WP_013527371.1	Cytochrome C nitrate reductase	1.8	5.12 × 10^−4^
WP_006995608.1	50S ribosomal protein L28	1.8	5.20 × 10^−14^
WP_006995166.1	F0F1 ATP synthase subunit gamma	1.8	8.24 × 10^−9^
WP_006995126.1	HU family DNA-binding protein	1.8	≤1.00 x 10^−17^
WP_006995592.1	Phosphopyruvate hydratase	1.8	≤1.00 x 10^−17^
WP_006995472.1	50S ribosomal protein L24	1.7	≤1.00 x 10^−17^
WP_013527660.1	Phenylalanine—tRNA ligase subunit alpha	1.7	2.22 × 10^−16^
WP_013525816.1	Universal stress protein UspE	1.7	2.68 × 10^−7^
WP_006995859.1	Uracil phosphoribosyltransferase	1.7	1.18 × 10^−9^
WP_005650775.1	Xanthine phosphoribosyltransferase	1.7	≤1.00 x 10^−17^
WP_006996581.1	Cytochrome bd oxidase subunit I	1.7	1.57 × 10^−13^
WP_005663485.1	Energy-dependent translational throttle protein EttA	1.6	3.03 × 10^−8^
WP_112130570.1	Type I glutamate—ammonia ligase	1.6	3.90 × 10^−4^
WP_006995866.1	Molecular chaperone DnaK	1.6	≤1.00 x 10^−17^
WP_005652337.1	2,3-diphosphoglycerate-dependent phosphoglycerate mutase	1.6	≤1.00 x 10^−17^
WP_006995159.1	Folate-binding protein YgfZ	1.6	7.89 × 10^−7^
WP_005626581.1	30S ribosomal protein S7	1.6	1.44 × 10^−10^
WP_006996015.1	Hypothetical protein	1.6	1.67 × 10^−3^
WP_150007580.1	Argininosuccinate synthase	1.6	3.71 × 10^−10^
WP_006996069.1	Extracellular solute-binding protein	1.6	≤1.00 x 10^−17^
WP_150007675.1	Elongation factor G	1.6	≤1.00 x 10^−17^
WP_006995605.1	Aspartate aminotransferase family protein	1.6	1.12 × 10^−3^
WP_150007737.1	Galactose-1-phosphate uridylyltransferase	1.5	3.96 × 10^−5^
WP_081457583.1	Phosphate ABC transporter substrate-binding protein PstS	1.5	2.60 × 10^−13^
WP_013525505.1	4-hydroxy-tetrahydrodipicolinate synthase	1.5	1.55 × 10^−10^
WP_005648164.1	Serine hydroxymethyltransferase	1.5	≤1.00 x 10^−17^
WP_013526528.1	N-acetylneuraminate lyase	1.5	2.44 × 10^−8^
WP_150007656.1	F0F1 ATP synthase subunit alpha	1.5	≤1.00 x 10^−17^
WP_005632756.1	50S ribosomal protein L23	2.5	1.82 × 10^−7^
WP_005650452.1	Stringent starvation protein A	2.4	2.79 × 10^−6^
WP_006995958.1	Phenylalanine—tRNA ligase subunit beta	2.3	≤1.00 x 10^−17^
Up regulated in ModA13-ON			
WP_005650820.1	Cell division protein ZapB	3.6	1.14 × 10^−4^
WP_005650804.1	Triose-phosphate isomerase	2.5	7.32 × 10^−6^
WP_005629492.1	Co-chaperone GroES	2.1	2.51 × 10^−6^
WP_005652591.1	Alkylphosphonate utilization protein	2.1	4.15 × 10^−3^
WP_080334623.1	Fe-S cluster assembly scaffold IscU	1.9	7.65 × 10^−4^
WP_013526254.1	YihD family protein	1.8	3.98 × 10^−7^
WP_150007641.1	Fe-S biogenesis protein NfuA	1.7	1.47 × 10^−8^
WP_006995574.1	OmpH family outer membrane protein	1.7	≤1.00 x 10^−17^
WP_005659903.1	Fe-S cluster assembly protein IscX	1.6	3.38 × 10^−3^
WP_032826317.1	Type I restriction endonuclease subunit R	1.5	1.28 × 10^−3^
WP_150007684.1	Peptidylprolyl isomerase	1.5	9.79 × 10^−6^
WP_005544465.1	Acyl carrier protein	1.5	1.10 × 10^−12^
WP_005689970.1	Thioredoxin	1.5	4.33 × 10^−5^

In the ModA16 ON-OFF pair, a total of 617 proteins were identified, representing a coverage of ~33% of the total proteome. Two hundred twenty proteins showed statistically significant changes in their abundance (adjusted *P*-value  <0.05) as a result of ModA16 expression. Twenty proteins exhibited a fold change ≥1.5-fold; eight of which were upregulated and 12 were downregulated in ModA16 ON relative to ModA16 OFF (Table 3). In ModA16 ON, there was an upregulation of a filamentous hemagglutinin N‐terminal domain‐containing protein (2.11-fold upregulation), matching that seen with our specific analysis of OMPs, and an upregulation of multiple iron-acquisition factors including an Iron ABC transporter substrate-binding protein (2.11-fold upregulation), and heme/hemopexin-binding proteins HxuC (2.09-fold upregulation), and HxuB (1.5-fold upregulation). Proteins downregulated in the ModA16 ON strain include those involved in metabolic pathways such as dehydrogenases and reductase enzymes, as well as a transcriptional regulator and an outer-membrane receptor (Table 3).

**TABLE 3:** Differential regulation of protein expression by the ModA16 phasevarion (>1.5-fold)

Accession	Protein	Fold change (ON vs OFF)	*p*-value
Down regulated in ModA16-ON			
WP_149998740.1	TonB-dependent receptor	1.7	≤1.00 x 10^−17^
WP_005694608.1	Anaerobic glycerol-3-phosphate dehydrogenase subunit C	1.7	6.94 × 10^−08^
WP_005653705.1	HTH-type transcriptional regulator CysB	1.6	1.42 × 10^−05^
WP_006995649.1	4-hydroxy-3-methylbut-2-enyl diphosphate reductase	1.6	1.07 × 10^−06^
WP_013526541.1	NADH:ubiquinone reductase (Na(+)-transporting) subunit B	1.6	≤1.00 x 10^−17^
WP_013526018.1	Phosphate acetyltransferase	1.5	≤1.00 x 10^−17^
WP_013526175.1	L-2,4-diaminobutyrate decarboxylase	1.5	4.77 × 10^−08^
WP_013526020.1	50S ribosomal protein L3 N(5)-glutamine methyltransferase	1.5	2.80 × 10^−14^
WP_013526174.1	Diaminobutyrate-−2-oxoglutarate transaminase	1.5	≤1.00 x 10^−17^
WP_013525731.1	LTA synthase family protein	1.5	1.87 × 10^−4^
WP_013525631.1	Exoribonuclease II	1.5	≤1.00 x 10^−17^
WP_013526011.1	Bifunctional protein-disulfide isomerase/oxidoreductase DsbC	1.5	≤1.00 x 10^−17^
			
Up regulated in ModA16-ON			
WP_11961812.1	Iron ABC transporter substrate-binding protein	2.1	≤1.00 x 10^−17^
WP_149998746.1	Filamentous hemagglutinin N-terminal domain-containing protein	2.1	≤1.00 x 10^−17^
WP_013525511.1	Heme/hemopexin-binding protein HxuC	2.1	01.25 × 10^−4^
WP_013525785.1	ShlB/FhaC/HecB family hemolysin secretion/activation protein	1.9	≤1.00 x 10^−17^
WP_013525630.1	Peptide chain release factor 3	1.5	7.39 × 10^−12^
WP_013525881.1	DNA topoisomerase IV subunit A	1.5	1.71 × 10^−4^
WP_013526463.1	DUF1523 family protein	1.5	3.58 × 10^−07^
WP_013525512.1	Heme/hemopexin-binding protein HxuB	1.5	2.40 × 10^−13^

### Serum killing

**Fig 3:**
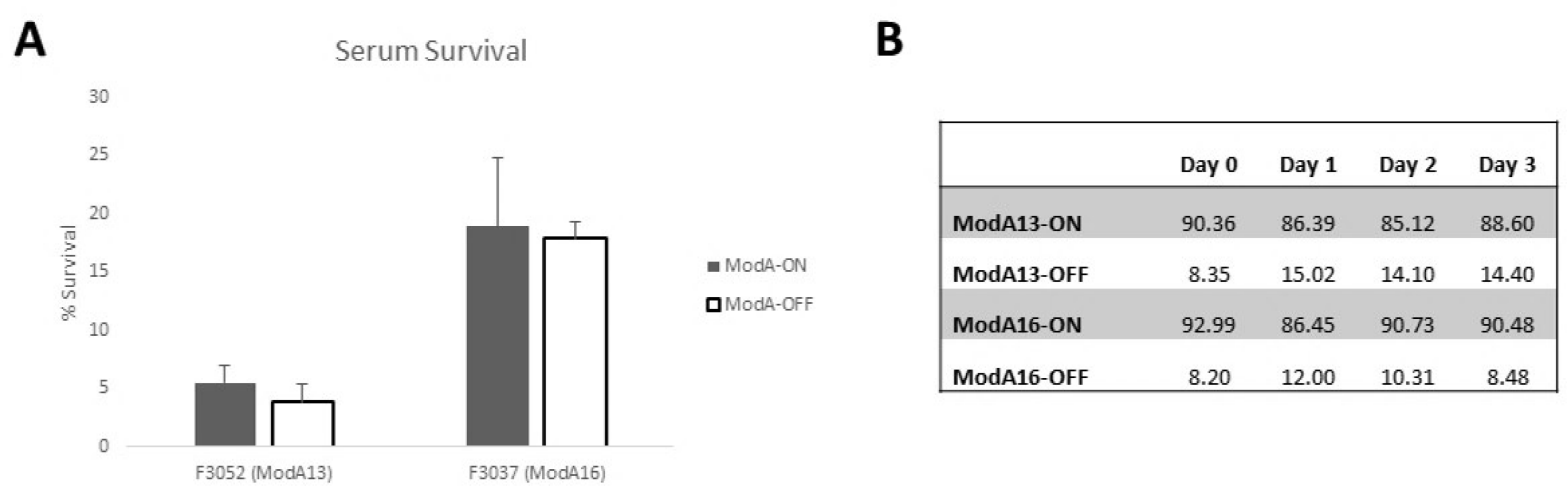
Assessment of serum survival in ModA containing strains of *H. influenzae* biogroup *aegyptius.* (**A**) Serum killing assays determined increased resistance to serum killing in BPF causing F3037 strains compared to non-BPF F3052 strains; however, no statistically significant difference was observed in populations enriched for ModA ON or ModA OFF in either strain. (**B**) Fragment length analysis of enriched *H. aegyptius* strains demonstrating the percentage of each population expressing ModA across successive rounds of serum killing. No statistically significant difference was observed in the ON-OFF status of each population over the course of the experiment. Experiments were carried in triplicate, with values presented in Table 3 representing a single biological replicate.

Previous literature suggests that there may be a trend for the BPF isolates to be more resistant to killing by normal human serum (NHS) (3). To determine whether ModA13 and/or ModA16 plays a role in this phenotype, and therefore could have a role in BPF, we used our enriched ModA13 (F3052) and ModA16 (F3037) ON-OFF pairs and monitored the response of these strains to NHS (Fig. 3A). A statistically significant difference was found in our assays between our F3052 (non-BPF) and F3037 (BPF) strains, with F3037 showing a significantly higher survival rate compared F3052, in line with previous literature (3). However, there was no statistically significant difference in resistance between populations enriched for the respective ModA ON and ModA OFF for each strain (Fig. 3A). F3052 enriched populations showed 5.5% survival for ModA13 ON and 3.9% for ModA13 OFF. F3037 populations demonstrated an 18.9% recovery for ModA16 ON and 17.9% for ModA16 OFF (Fig. 3A).

Although there was no significant difference in NHS survival of each isogenic ON vs OFF pair, there was a small difference in percent survival when comparing the isogenic ON vs OFF pairs. Therefore, these small difference in survival (ModA13 ON 5.5% vs OFF 3.9%; ModA16 ON 18.9% vs OFF 17.9%) could, over time, lead to a selection for a particular ModA state if one gives a small advantage to survival, potentially shifting strains toward more resistant phenotypes. Therefore, we subjected each ON-OFF strain pair to repeated NHS killing assays. Following the first round of serum killing, all surviving bacteria were harvested from plates, input OD normalized to an A_600_ of 0.02, and used as the input in the next assay. This was repeated for three rounds of NHS killing/survival. At each stage (input and output), the ON/OFF status of the *modA* gene was assessed using our fragment length analysis PCR strategy as used above to enrich these strains (Fig. 1). Despite multiple rounds of serum killing, no significant change in population percentages was observed (Fig. 3B), indicating no selection for particular ModA13 or ModA16 ON/OFF states is occurring. This is indicative that the genes regulated may not be providing an advantage during pathobiology, and certainly do not play a role in survival in normal human serum *in vitro*, a key phenotype of BPF strains.

## DISCUSSION

To date, all previously described phasevarions in human-adapted pathogens regulate the expression of virulence-associated factors (19, 26, 32, 33). Using strains which we enriched for defined SSR tract lengths, we demonstrated that both ModA13 and ModA16 are phase-variable, commensurate with previous studies on different *modA* alleles (20, 22, 23). SMRT sequencing and methylome analysis of our ModA16 ON-OFF pair and plasmid DNA from *E. coli* over-expressing the ModA16 protein demonstrated that the ModA16 methyltransferase recognizes the motif 5′-GGRC^(**m6**)^ **A**, demonstrating that the expressed ModA16 actively methylates DNA.

We also confirmed that the ModA13 recognition motif is GCAC^(m6)^ **A**, the same motif as detected in *N. gonorrhoeae* expressing this methyltransferase (29). These findings contradict the earlier motif described for ModA13, which was determined using restriction-inhibition assays (22). However, the motif of GCAC^(m6)^ **A** was present in the DNA probe used to determine the now incorrect ACAA^(m6)^ **A** motif (22), as GCACAAA [the last three bp of the five bp GCACA motif overlaps (ACA) the first three bp of the now incorrect ACAAA motif], which in turn was overlapped by the ApoI RAATTY restriction site used to originally call the ModA13 motif as ACAA^(m6)^ **A** (22). This perhaps illustrates the deficiencies in using restriction-inhibition assays to determine methyltransferase specificities.

As both ModA13 and ModA16 methyltransferases are phase-variable, and both methylate distinct target motifs, we determined that expression of numerous proteins are controlled by the resulting differential methylation, i.e., ModA13 and ModA16 control different phasevarions. When ModA13 is ON, the majority of proteins which demonstrate variable expression are ribosomal and metabolic proteins. Outer-membrane proteins are frequently major virulence determinants (34) and OmpH was upregulated in response to ModA13. The restriction subunit of a Type I R-M system was also found to be upregulated; these systems have been frequently found to play a large role in an alternate form of epigenetic phase variation (17). This may pose an additional form of complexity to phase-variable gene regulation as expression of a component of a R-M system is itself regulated by the phase-variable ModA13 system.

When ModA16 is ON, multiple members of the Hxu family of proteins, involved in heme acquisition and virulence (35), were upregulated. In addition, a hemolysin subunit and a protein containing the N-terminal domain of filamentous hemagglutinin (36) were also upregulated in ModA16 ON. Hemagglutination in particular is a process that has been previously identified as being important to the development of BPF (37). Proteins containing the N-terminal domain of filamentous hemagglutination can be found in large adhesins like the HMW proteins, high molecular weight protein adhesins, found in related *Haemophilus* species (38), and which also play major roles in virulence. As the iron acquisition and hemagglutination are processes which typically play a crucial role in virulence (34) it is tempting to speculate that they have a role in the manifestation of BPF, but this would require extensive experimental validation. When ModA16 is OFF, the levels of a protein involved in membrane transport were significantly increased (OMP analysis), as were multiple proteins involved in central metabolism. It could be that upregulation of these proteins in ModA16 OFF results in increased growth rate or increased survival in particular host niches, but the lack of a suitable animal model for BPF, and the difficulty in manipulating *H. influenzae* biogroup *aegyptius* genetically (6) means that confirming this is difficult to confirm experimentally.

Currently, little is known about what distinguishes BPF causing and non-BPF causing strains of *H. influenzae* biogroup *aegyptius*. Increased resistance to killing by human serum is a factor previously determined to be a characteristic of BPF vs non-BPF strains (3). As the ModA16 phasevarion is unique to only BPF causing isolates of *Haemophilus influenzae* biogroup *aegyptius*, it was hypothesized that ModA16 may contribute to the development of BPF through resistance to serum killing. Though our data trends do support previous experimental evidence that BPF causing strains of *H. influenzae* biogroup *aegyptius* are more resistant to serum killing (3), ModA16 does not appear to influence this resistance as ModA16 ON and ModA16 OFF strains do not show a significant difference in survival. Serum survival also does not appear to exert a selective pressure on populations of *H. influenzae* biogroup *aegyptius* as no significant shifts were seen in populations of ModA16 ON or ModA16 OFF strains upon subsequent rounds of serum killing. These findings were also observed in enriched populations of ModA13, which is not exclusive to BPF causing strains of *H. influenzae* biogroup *aegyptius*. Previous work also hypothesized that lipopolysaccharide (LPS) is involved in BPF development (39), with sialic acid of the LPS contributing to serum resistance in *H. influenzae* (40). Perhaps, a combination of unique LPS structure plus differential regulation of multiple surface proteins, including those controlled by the ModA16 phasevarion, are all involved in BPF development.

Phasevarions alter the expression of multiple genes and regulate proteins involved in virulence as well as proteins which are investigated as antimicrobial targets. Our characterization of the ModA16 phasevarion in *H. influenzae* biogroup *aegyptius* confirms previous assumptions that BPF is not the result of regulation of a single bacterial factor (4). It is likely that complex regulation of multiple bacterial proteins causes the unusual phenotype seen in a subset of *H. influenzae* biogroup *aegyptius* strains, and further experimental work is required to identify these factors.

## MATERIALS AND METHODS

### Bacterial growth conditions

*Haemophilus influenzae* biogroup *aegyptius* strains were grown in supplemented Brain Heart Infusion [sBHI; BHI media (Oxoid) plus nicotinamide adenine dinucleotide (1% vol/vol) and hemin (2 µg/mL)] broth or sBHI agar (sBHI broth with 1.5% wt/vol bacteriological agar). Agar plates were incubated for 24 hours at 37°C under 5% (vol/vol) CO_2_, liquid cultures were incubated overnight at 37°C with shaking.

*Escherichia coli* was grown at 37°C in lysogeny broth at 200 rpm as standard. Where required, bacteriological agar was added to 1.5% (wt/vol) to prepare solid media. To maintain the pET28a vector, kanamycin was added at 50 µg/mL where appropriate.

### Cloning and over-expression of ModA16

PCR products generated for cloning into the NdeI/BamHI site of pET28a cloning vector (EMD Millipore) were prepared using KOD Hot-start DNA polymerase (EMD Millipore) according to manufacturer’s instructions, using *H. influenzae* biogroup *aegyptius* strain F3037 genomic DNA. Primers specific for the *modA16* were (forward: 5′-AGTCAG **CATATG** AAGACAGACATTCAAACCG-3′; reverse: 5′-AGTCAG **GGATCC** TCATTCGCCATCTTTTTTCTCCG-3′). NdeI and BamHI sites are highlighted in **bold** text. BL21 containing only pET28a was used as a control, in a similar strategy as used previously (20). Over-expression of the ModA16 protein was carried out using *E. coli* BL21 cells, which were induced by the addition of isopropyl β-D-1-thiogalactopyranoside (IPTG) to a final concentration of 0.5 mM overnight at 37°C with shaking at 200 rpm. Plasmid DNA for SMRT sequencing was isolated from these overnight-induced cells using a plasmid mini prep kit (Qiagen) as per manufacturer’s instructions.

### Strain enrichment

A FAM-labeled PCR amplifying across the AGCC_(n)_ repeat tract of *modA13* and *modA16* coupled with fragment length analysis was used to determine the length of this SSR tract length in representative strains of *H. influenzae* biogroup *aegyptius* (strains F3052 for *modA13*, F3037 for *modA16*). Primers Him1F (5′-FAM-ATGGCGGACAAAGCACCGAAGG-3′) and Him3 (5′-CAAAAAGCCGGTCAATTTCATCAAA-3′) were used as described previously (18, 20). DNA fragment length analysis was carried out at the Australian Genome Research Facility (Brisbane, Australia). For enrichment of ModA ON and OFF strains, stocks of each prototype *H. influenzae* biogroup *aegyptius* strain were plated out to single colonies and fragment length analysis was used to assess SSR tract lengths of each colony. Colonies that contained the desired tract length were sub-cultured and the process was repeated until strains were enriched for the desired tract length of approximately 90%. F3052 ModA13 ON strains were enriched for 13 AGCC repeats and ModA13 OFF strains were enriched for 12 AGCC repeats. F3037 ModA16 ON strains were enriched for 10 AGCC repeats and ModA16 OFF strains were enriched for 9 AGCC repeats.

### SMRT sequencing and methylome analysis

Genomic DNA from our enriched ON-OFF pairs of *H. influenzae* biogroup *aegyptius* strain F3052 (ModA13) and F3037 (ModA16) were prepared from an overnight culture in sBHI broth and high molecular weight genomic DNA was isolated using the Sigma Genelute kit (Sigma Aldrich) according to the manufacturer’s instructions. Plasmid DNA from *E. coli* over-expressing ModA16 from pET28a, or containing pET28a only, was prepared using the Qiagen mini prep kit according to manufacturer’s instructions. SMRT sequencing and methylome analysis was carried out as previously described (41, 42). Briefly, DNA was sheared to an average length of approximately 5–10 kb (genomic DNA) using g-TUBEs (Covaris) and SMRTbell template sequencing libraries were prepared using sheared DNA. DNA was end repaired, then ligated to hairpin adapters. Incompletely formed SMRTbell templates were degraded with a combination of Exonuclease III (New England Biolabs) and Exonuclease VII (USB; Cleveland, OH, USA). Primer was annealed and samples were sequenced on the PacBio Sequel system (Menlo Park, CA, USA) using standard protocols for long insert libraries. SMRT sequencing and methylome analysis was carried out at SNPSaurus (University of Oregon, USA).

### SDS-PAGE and Western blot

Enriched ON-OFF pairs of each representative strain of *H. influenzae* biogroup *aegyptius* were harvested from sBHI media into phosphate buffered saline (PBS), and the optical density (OD)_600_ was normalized to 5.0. These suspensions were prepared for SDS-PAGE with BOLT 4× loading dye containing 100 mM dithiothreitol (Sigma-Aldrich) and boiled for 30 min before centrifugation at 12,000 *× g* for 15 min. Samples were loaded on precast 4%–12% NuPAGE Bis-Tris gel (Invitrogen) and run for 45 min at 165V in 3-(N-morpholino)propanesulfonic acid (MOPs) buffer (Thermo Fisher). Proteins were visualized either by Coomassie brilliant blue staining or silver stained using a ProteoSilver silver stain kit (Sigma-Aldrich). Western blot analysis using anti-ModA antisera was performed as previously described (20). All protein gels and Westerns were scanned using an HP scanner to produce a Hi-Res TIFF format image.

### Mass spectrometry analysis of ModA ON-OFF outer-membrane proteins

Overnight cultures of *H. influenzae* biogroup *aegyptius* enriched ON-OFF strain pairs were harvested from sBHI and resuspended in 4.5 mL 10 mM Tris pH 8. Samples were then sonicated twice for 30-second bursts until the suspension was transparent. Samples were cleared by centrifugation at 4,500 rpm for 5 min to remove unlysed cells. Four milliliters of a 2% sarkosyl (Sigma) solution was added to the supernatant and mixed well. Samples were centrifuged for 1.5 hours at 35,000 rpm in an Optima L100XP ultracentrifuge using a 90Ti rotor (Beckman Coulter). The supernatant was removed and the pellet was resuspended in 4 mL 10 mM Tris pH 8 before the addition of 4 mL 2% (wt/vol) sarkosyl. Centrifugation was repeated and the supernatant was removed and the pellet resuspended in 10 mM Tris pH 8.0.

Concentration of OMPs were normalized following a bicinchoninic acid (BCA) protein assay according to manufacturer’s instructions (Thermo Fisher) and assessed with Coomassie-stained SDS-PAGE as above. The protein was then separated in 4%–12% Bis-Tris SDS-PAGE gel and stained with Coomassie blue. The bands of interest were excised and reduced and alkylated by 10 mM dithiothreitol (DTT) and 25 mM acrylamide, respectively. In-gel trypsin digestion was performed. Tryptic digested peptides were analyzed using a Shimadzu Prominence nanoHPLC system (Shimadzu Corp) and 5,600 Triple TOF mass spectrometer (SCIEX), and data were processed and analyzed by Protein-Pilot software (SCIEX) as previously described (43).

### Whole cell SWATH-MS

Duplicate overnight cultures of each enriched ON-OFF pair of representative *H. influenzae* biogroup *aegyptius* strains (10^7^ CFU/mL) were harvested from sBHI. The cultures were lysed in urea buffer containing 8 M urea, 50 mM ammonium bicarbonate, and 5 mM DTT and then incubated at 56°C for 30 min. The cysteines of the total protein were alkylated by addition of acrylamide to a final concentration of 25 mM and incubated at room temperature for 30 min in the dark. Subsequently, the samples were diluted with 50 mM ammonium bicarbonate to reduce urea to 2 M. For trypsin digestion, trypsin (New England Biolabs) was added to samples at a 1:100 enzyme to protein ratio, and the mixture was incubated at 37°C overnight. The resulting tryptic digested peptides were then desalted and purified using a Ziptip (Millipore) as per manufacturer instructions. SWATH-MS analysis was conducted using a liquid chromatography-tandem mass spectrometry comprising a Prominence nanoLC system (Shimadzu) and Triple TOF 5600 instrument with a NanoSpray III interface (SCIEX), following a previously described method (44). Protein identification was performed using ProteinPilot 5.1 (SCIEX) with the search conducted against the *H. influenzae* biogroup *aegyptius* F3052 genome (NCBI Accession CP043810.1) or the F3037 genome (NCBI Accession CP043772.1). The results obtained from ProteinPilot were used as an ion library for quantifying the abundance of peptides and proteins using PeakView 2.1 (SCIEX) with standard setting. Comparison of protein relative abundance was performed based on protein intensities or ion intensities using a linear mixed-effects model with the MSstats package in R. Proteins with adjusted *P*-values of <0.05 were considered significant. The mass spectrometry proteomics data have been deposited to the ProteomeXchange Consortium via the PRIDE (45) partner repository, and the data set identifier is PXD036097.

### Serum killing assays

Overnight growth of enriched *H. influenzae* biogroup *aegyptius* ON-OFF strain pairs was harvested from sBHI plates and was used to inoculate sBHI broth and incubated at 37°C for 4–6 hours. Broth cultures were then harvested via centrifugation, washed once with Hank’s balanced salt solution (HBSS), and resuspended to an A_600_ of 0.2. Ten microliters of the bacterial suspension (~2.0 × 10^4^ cfu) was added to a final volume of HBSS containing 16.6% normal human serum (Sigma-Aldrich) and 10% rabbit complement (Sigma-Aldrich). HBSS only, serum only, and complement only were included as controls. HBSS assays were incubated at 37°C for 1 hour prior to enumeration on supplemented BHI plates. All input cultures were also enumerated prior to incubation, and survival was calculated as a percentage of the corresponding input cfu.

## Data Availability

The mass spectrometry proteomics data have been deposited to the ProteomeXchange Consortium via the PRIDE (45) partner repository, and the data set identifier is PXD036097.

## SUPPLEMENTAL MATERIAL

Data S1 spectrum.02601-23-s0001.xlsx Methylome data for all methyltransferases.

Figure S1 spectrum.02601-23-s0002.pdf ModA expression in enriched populations of *H. influenzae* biogroup aegyptius.

Figure S2 spectrum.02601-23-s0003.pdf Full Coomassie-stained SDS PAGE and anti-ModA Western blot demonstrating over-expression of ModA16 heterologously in *E. coli* BL21 (DE3) from the pET28a vector.

ASM does not own the copyrights to Supplemental Material that may be linked to, or accessed through, an article. The authors have granted ASM a non-exclusive, world-wide license to publish the Supplemental Material files. Please contact the corresponding author directly for reuse.
