# Supplementary material for: Phasevarions in Haemophilus influenzae biogroup aegyptius control expression of multiple proteins
Source: Microbiol Spectr. 2023 Dec 6;12(1):e02601-23. doi: 10.1128/spectrum.02601-23 (PMC10783040; doi:10.1128/spectrum.02601-23)
Supplement: Figure S1 — ModA expression in enriched populations of H. influenzae biogroup aegyptius. [file spectrum.02601-23-s0002.pdf]

Supplementary Figure 1

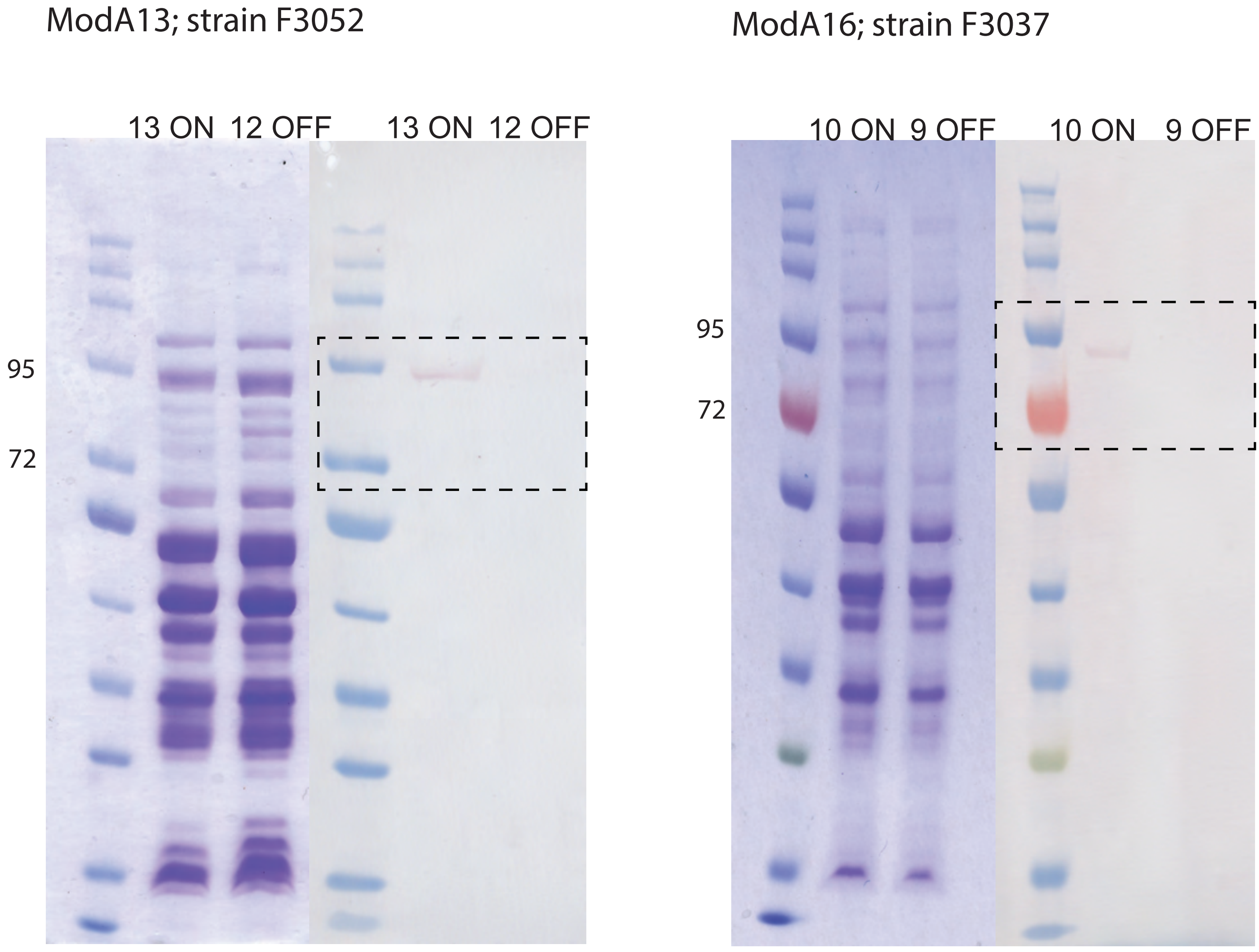

Supplementary Figure 1 – ModA expression in enriched populations of H. influenzae biogroup aegyptius. Coomassie staining of whole cell lysates of enriched ModA13-ON (RT13) and ModA13-OFF (RT12) populations of strain F3052 (A). Western shows that ModA13 is only expressed in populations enriched for a AGCC(13) repeats (B). Coomassie staining of whole cell lysates of F3037 populations enriched for ModA16- ON (RT10) and ModA16- OFF (RT9) (C). Western blot demonstrates that ModA16 is only expressed in populations enriched for AGCC(10) repeats (D).
