# Supplementary material for: Phasevarions in Haemophilus influenzae biogroup aegyptius control expression of multiple proteins
Source: Microbiol Spectr. 2023 Dec 6;12(1):e02601-23. doi: 10.1128/spectrum.02601-23 (PMC10783040; doi:10.1128/spectrum.02601-23)
Supplement: Figure S2 — Full Coomassie-stained SDS PAGE and anti-ModA Western blot demonstrating over-expression of ModA16 heterologously in E. coli BL21 (DE3) from the pET28a vector. [file spectrum.02601-23-s0003.pdf]

Supplementary Figure 2

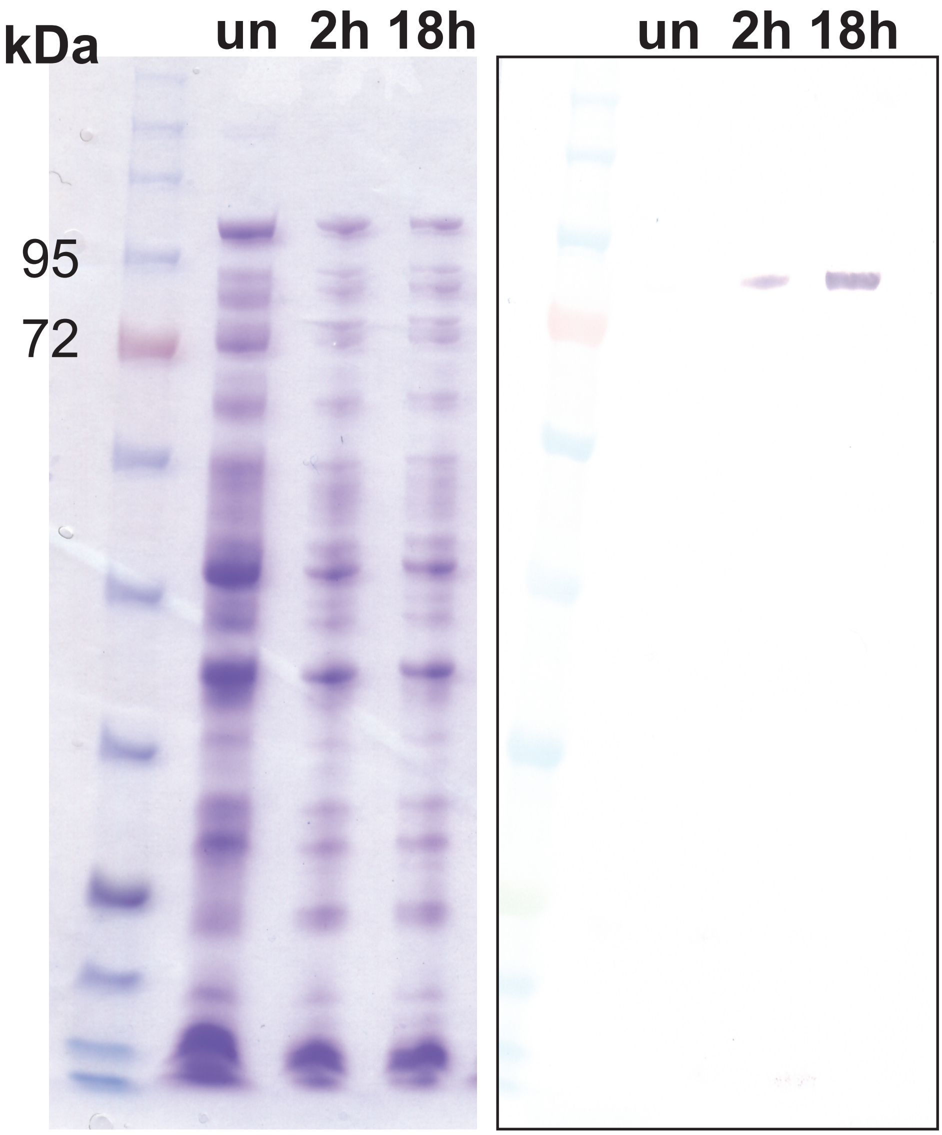

Full coomassie stained SDS PAGE and anti-ModA Western blot demonstrating over-expression of ModA16 heterologously in E. coli BL21 (DE3) from the pET28a vector. Cultures griwn for 18hrs were used to prepare plasmid DNA for SMRT sequencing and methylome analysis
